# Proceedings of the Ninth Annual Deep Brain Stimulation Think Tank: Advances in Cutting Edge Technologies, Artificial Intelligence, Neuromodulation, Neuroethics, Pain, Interventional Psychiatry, Epilepsy, and Traumatic Brain Injury

**DOI:** 10.3389/fnhum.2022.813387

**Published:** 2022-03-04

**Authors:** Joshua K. Wong, Günther Deuschl, Robin Wolke, Hagai Bergman, Muthuraman Muthuraman, Sergiu Groppa, Sameer A. Sheth, Helen M. Bronte-Stewart, Kevin B. Wilkins, Matthew N. Petrucci, Emilia Lambert, Yasmine Kehnemouyi, Philip A. Starr, Simon Little, Juan Anso, Ro’ee Gilron, Lawrence Poree, Giridhar P. Kalamangalam, Gregory A. Worrell, Kai J. Miller, Nicholas D. Schiff, Christopher R. Butson, Jaimie M. Henderson, Jack W. Judy, Adolfo Ramirez-Zamora, Kelly D. Foote, Peter A. Silburn, Luming Li, Genko Oyama, Hikaru Kamo, Satoko Sekimoto, Nobutaka Hattori, James J. Giordano, Diane DiEuliis, John R. Shook, Darin D. Doughtery, Alik S. Widge, Helen S. Mayberg, Jungho Cha, Kisueng Choi, Stephen Heisig, Mosadolu Obatusin, Enrico Opri, Scott B. Kaufman, Prasad Shirvalkar, Christopher J. Rozell, Sankaraleengam Alagapan, Robert S. Raike, Hemant Bokil, David Green, Michael S. Okun

**Affiliations:** ^1^Department of Neurology, Fixel Institute for Neurological Diseases, University of Florida, Gainesville, FL, United States; ^2^Department of Neurology, Christian-Albrechts-University, Kiel, Germany; ^3^Department of Medical Neurobiology (Physiology), Institute of Medical Research Israel-Canada, Hebrew University of Jerusalem, Jerusalem, Israel; ^4^Biomedical Statistics and Multimodal Signal Processing Unit, Section of Movement Disorders and Neurostimulation, Focus Program Translational Neuroscience, Department of Neurology, University Medical Center of the Johannes Gutenberg-University Mainz, Mainz, Germany; ^5^Department of Neurological Surgery, Baylor College of Medicine, Houston, TX, United States; ^6^The Human Motor Control and Neuromodulation Laboratory, Department of Neurology and Neurological Sciences, Stanford University School of Medicine, Stanford University, Stanford, CA, United States; ^7^Department of Neurological Surgery, Kavli Institute for Fundamental Neuroscience, University of California, San Francisco, San Francisco, CA, United States; ^8^Department of Anesthesia, University of California, San Francisco, San Francisco, CA, United States; ^9^Department of Neurology, Wilder Center for Epilepsy Research, University of Florida, Gainesville, FL, United States; ^10^Department of Neurology, Mayo Clinic, Rochester, NY, United States; ^11^Department of Neurosurgery, Mayo Clinic, Rochester, NY, United States; ^12^Department of Neurology, Weill Cornell Brain and Spine Institute, Weill Cornell Medicine, New York, NY, United States; ^13^Department of Neurosurgery, Stanford University, Stanford, CA, United States; ^14^Department of Electrical and Computer Engineering, University of Florida, Gainesville, FL, United States; ^15^Department of Neurosurgery, Fixel Institute for Neurological Diseases, University of Florida, Gainesville, FL, United States; ^16^Queensland Brain Institute, University of Queensland and Saint Andrews War Memorial Hospital, Brisbane, QLD, Australia; ^17^National Engineering Laboratory for Neuromodulation, School of Aerospace Engineering, Tsinghua University, Beijing, China; ^18^Department of Neurology, Faculty of Medicine, Juntendo University, Tokyo, Japan; ^19^Neuroethics Studies Program, Department of Neurology, Georgetown University Medical Center, Washington, DC, United States; ^20^US Department of Defense Fort Lesley J. McNair, National Defense University, Washington, DC, United States; ^21^Department of Philosophy and Science Education, University of Buffalo, Buffalo, NY, United States; ^22^Department of Psychiatry, Massachusetts General Hospital and Harvard Medical School, Boston, MA, United States; ^23^Department of Psychiatry, University of Minnesota, Minneapolis, MN, United States; ^24^Department of Neurology and Neurosurgery, Icahn School of Medicine at Mount Sinai, New York, NY, United States; ^25^Department of Neurology, Emory University, Atlanta, GA, United States; ^26^Department of Psychology, Columbia University, New York, NY, United States; ^27^Department of Anesthesiology (Pain Management) and Neurology, University of California, San Francisco, San Francisco, CA, United States; ^28^School of Electrical and Computer Engineering, Georgia Institute of Technology, Atlanta, GA, United States; ^29^Restorative Therapies Group Implantables, Research and Core Technology, Medtronic Inc., Minneapolis, MN, United States; ^30^Boston Scientific Neuromodulation Corporation, Valencia, CA, United States; ^31^NeuroPace, Inc., Mountain View, CA, United States

**Keywords:** deep brain stimulation, artificial intelligence, neuroethics, pain, interventional psychiatry, adaptive DBS, epilepsy, traumatic brain injury

## Abstract

DBS Think Tank IX was held on August 25–27, 2021 in Orlando FL with US based participants largely in person and overseas participants joining by video conferencing technology. The DBS Think Tank was founded in 2012 and provides an open platform where clinicians, engineers and researchers (from industry and academia) can freely discuss current and emerging deep brain stimulation (DBS) technologies as well as the logistical and ethical issues facing the field. The consensus among the DBS Think Tank IX speakers was that DBS expanded in its scope and has been applied to multiple brain disorders in an effort to modulate neural circuitry. After collectively sharing our experiences, it was estimated that globally more than 230,000 DBS devices have been implanted for neurological and neuropsychiatric disorders. As such, this year’s meeting was focused on advances in the following areas: neuromodulation in Europe, Asia and Australia; cutting-edge technologies, neuroethics, interventional psychiatry, adaptive DBS, neuromodulation for pain, network neuromodulation for epilepsy and neuromodulation for traumatic brain injury.

## Introduction

The DBS Think Tank IX presenters pooled data and determined that DBS expanded in its scope and has been applied to multiple brain disorders in an effort to modulate neural circuitry. It was estimated that globally more than 230,000 deep brain stimulation (DBS) devices have been implanted for neurological and neuropsychiatric disorders. The DBS Think Tank was founded in 2012 and it provides an open platform where clinicians, engineers and researchers (from industry and academia) can freely discuss current and emerging DBS technologies as well as the logistical and ethical issues facing the field. The emphasis of the DBS Think Tank is on cutting edge research and collaboration with the potential to advance the DBS field. The DBS Think Tank IX was held on August 25–27 in Orlando FL with US based participants largely in person and overseas participants joining by video conferencing technology. The meeting was focused on advances in the following areas: neuromodulation in Europe, Asia and Australia; cutting-edge technologies, neuroethics, interventional psychiatry, adaptive DBS, neuromodulation for pain, network neuromodulation for epilepsy and neuromodulation for traumatic brain injury. The DBS Think Tank discussed Maslow’s theories and a path to transcendence both for patients as well as for DBS practitioners. The attendees also participated in a DBS Think Tank survey, which documented the expansion of DBS into several indications such as movement disorders, psychiatric disorders, and pain disorders. This proceeding summarizes the advances discussed at the DBS Think Tank IX.

## International Neuromodulation Trends From Europe, Asia and Australia

### Clinical Predictors of the Deep Brain Stimulation Effect on Parkinson’s Disease

**FIGURE 1:**
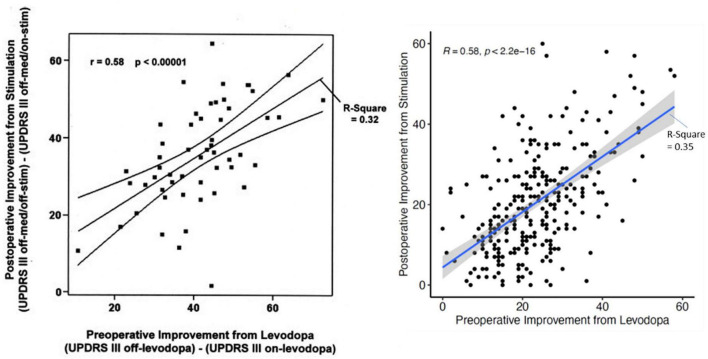
The relation of the UPDRS III-improvement during the preoperative L-dopa-test and the postoperative improvement of the UPDRS III-score due to stimulation only for the first description in 2002 (Charles et al.; *n* = 56 patients) and current data from Kiel (*n* = 334 patients) with almost the same statistical relations. Nevertheless, the dispersion for individual patients is very large.

Individualization of treatment for persons with Parkinson’s disease (PD) is among the main objectives of neurology. The response to treatment is heterogenous and critically depends on our ability to predict the response of a particular patient to different interventions. For DBS we take for granted that the response of a patient to levodopa best predicts the response to stimulation (Charles et al., 2002). But while we can confirm this in our cohort (*n* = 334 patients; *R*: 0.58; *R*^2^: 0.35; *p* < 2.2e^–16^) the response of the individual patient can vary considerably (Figure 1). Two approaches may address the variability: (1) First is the application of new statistical machine learning technique(s). Generalized linear models using clinical and medical history data can be used. Following appropriate cross validation, the prediction improves to a maximum mean-*R*^2^ of 0.358. When outcomes are dichotomized (e.g., UPDRS III-score improvement > 33%) and advanced modeling and machine learning is applied, the best discriminators approximated an AUC of 0.65. Better fits could be obtained with restricted criteria (Habets et al., 2020). Although promising, much larger, pooled patient cohorts will be required to determine the limits of this methodology (Habets et al., 2020). The second approach will be to use more specific and dichotomized outcomes (e.g., tremor severity, quality of life, freezing) for prediction. This approach leads to improvement in the AUC of 0.86 for freezing or balance and improvement to 0.75 for quality of life (Gavriliuc et al., 2021; Jost et al., 2021; Yin et al., 2021). The second approach has more latitude for improvement.

### Optimizing the Asleep Deep Brain Stimulation Surgical Procedure

DBS is currently a standard procedure for advanced PD. DBS is not only used for PD patients but can be applied for patients with other movement disorders including dystonia, essential tremor (ET) and psychiatric disorders. The wide range of applications suggests that enhancement of this technique could be far reaching. Many centers employ awake physiological navigation and stimulation assessments to optimize DBS localization and outcome. However, many patients remain fearful of the awake brain surgery, leaving a wide gap for therapeutic improvement.

To enable DBS under sedation, asleep DBS, we characterized the cortico-basal ganglia neuronal network of two non-human primates under propofol, ketamine and interleaved propofol-ketamine (IPK) sedation (Guang et al., 2021). Further, we compared these sedation states in the healthy and Parkinsonian condition to those of healthy sleep.

**FIGURE 2:**
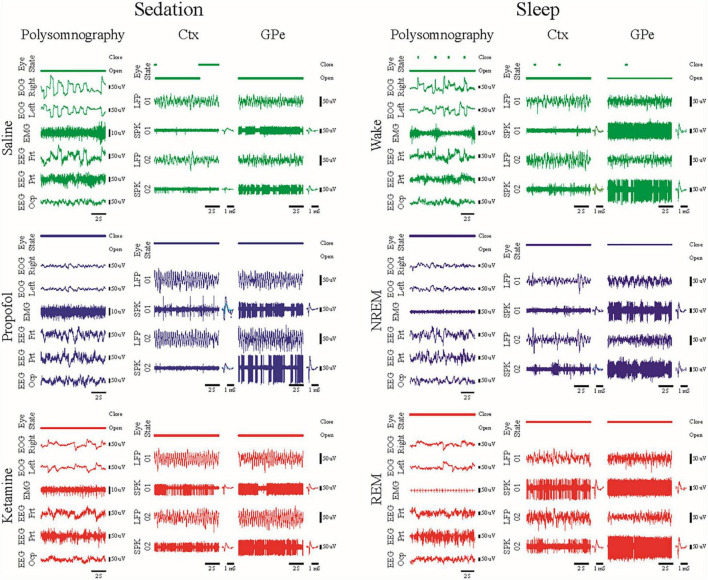
Examples of 10 s traces of polysomnography (eye open/close state, EOG, EMG and EEG), local field potential and spiking activity (LFP/SPK) recorded in the frontal cortex and the external segment of the globus pallidus (Ctx/GPe) during sedation sessions (left, saline baseline—upper, green; propofol—center, blue; and ketamine—lower, red) and during the awake-sleep cycle (right, wake—upper, green; NREM—center, blue; and REM—lower, red). Modified with permission from Guang et-al 2021 (Guang et al., 2021).

We found in polysomnography and neuronal activity recordings in animals treated with ketamine increases high-frequency power and synchronization while propofol increases low-frequency power and synchronization (Figure 2). Thus, ketamine does not mask the low-frequency oscillations used for physiological navigation toward basal ganglia DBS targets. The brain spectral state under ketamine and propofol mimicked rapid eye movement (REM) and non-REM (NREM) sleep activity, respectively, and the IPK protocol resembled the fast dynamics of the NREM-REM sleep cycle.

These promising results in animal models may be the first step toward asleep DBS with non-distorted physiological navigation. The clinical outcome and the subjective evaluation of the patients under the IPK sedation protocol should be tested in open and then prospective double-blind studies.

### Fusing Electrophysiology and Neuroimaging for Optimal Deep Brain Stimulation

**FIGURE 3:**
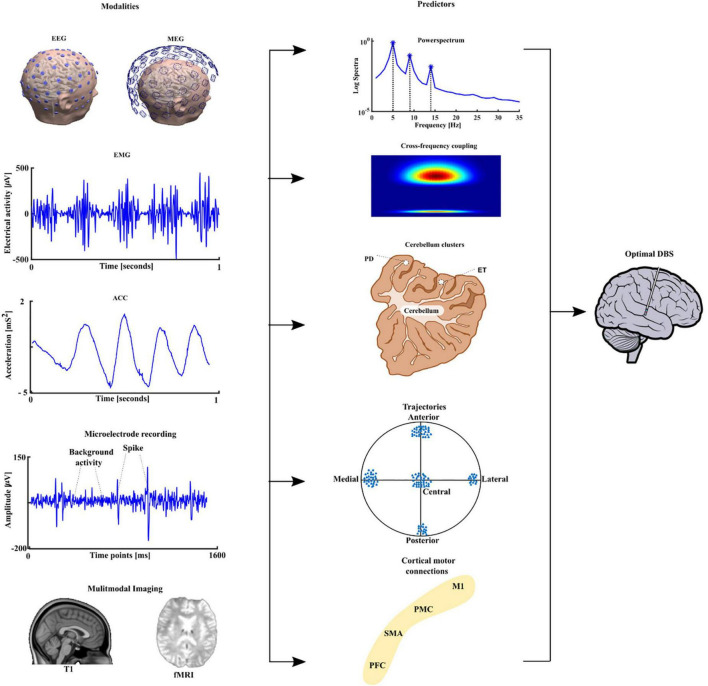
The different modalities are depicted in the first column namely the electroencephalography (EEG), magnetoencephalography (MEG) followed by peripheral signals namely electromyography (EMG), accelerometer (ACC), microelectrode recording (MER), and neuroimaging namely structural T1 magnetic resonance imaging and functional magnetic resonance imaging (fMRI). The extracted predictors are shown in the second column power spectrum, cross-frequency coupling, source analyses-based cerebellum clusters for PD and ET. Trajectory based oscillatory predictors and finally cortical motor connectivity with the four main motor regions, primary motor cortex (M1), premotor cortex (PMC), supplementary motor area (SMA) and prefrontal cortex (PFC) to sub thalamic nucleus (STN). The multimodal integration of all these features can lead to optimal deep brain stimulation in PD patients.

The identification of functional predictors using brain imaging and electrophysiological techniques is essential for improving direct planning of DBS implantation, but also for advancing developments in neuromodulation. One way to achieve this goal will be through the development of the fusion of multimodal imaging and advanced data analyses techniques from electrophysiological pre- and intraoperative recordings, that support electrode placement during the stereotactic procedure and the postoperative programming (Gonzalez-Escamilla et al., 2020; Muthuraman et al., 2020). This is particularly relevant when considering electrophysiological data in tremor patients, using peripheral signals namely electromyography (EMG) and accelerometer (ACC) as vital techniques to assess the suitability for DBS treatment. We started by looking at the mean harmonic power of the accelerometer recordings in tremor patients carrying out a holding condition. We were able to distinguish between PD and ET patients with 94% accuracy and proposed this measure as a new diagnostic test (Muthuraman et al., 2011). Recently, we have developed measures like the tremor stability index and we compared them with the mean harmonic power and some new measures like cross frequency coupling between two EMG or ACC signals from different muscles (di Biase et al., 2017). To identify direct electrophysiological signatures which relate to these findings from the periphery, simultaneous measurement of electroencephalography (EEG) will be required. However, EEG recorded in patients receiving DBS induces artifacts in the frequency domain of the signals, namely at the frequency of the stimulation (130 or 160 Hz) and subsequent harmonics. To examine the frequency signatures, first the artifact needs to be removed. Instead of a simple low pass filter which takes all the information above the cut-off frequency, we were able to develop a method which takes into account both time and frequency domain dynamics (Santillán-Guzmán et al., 2013). Once the artifacts were removed, the oscillatory features could be estimated from the EEG signals, or local field potentials (LFP), which our group (Muthuraman et al., 2018; Tamás et al., 2018) and many other groups have shown as robust predictors (Litvak et al., 2021). We applied microelectrode recording and looked at the optimal predictor for subthalamic nucleus (STN) targeting and optimal trajectories and we were able to show both beta and gamma oscillatory activity as good predictors (Koirala et al., 2020). After identifying robust predictors, further work to translate the predictors to the clinical setting is ongoing. This process is outlined in Figure 3. We see a clear need for the multimodal integration of distinct features and models to tune and to understand the pathophysiological DBS mechanisms. In addition, models for the long-term outcome prediction and improvement or real-time electrophysiological monitoring will be useful. These models should be easily introduced into clinical practice and should guide our focus of future directions, inclusive of sensor engineering.

### Updates on Deep Brain Stimulation for Tourette’s Syndrome in Australia

In our Neuroscience Centre (Neurosciences Queensland), DBS of the anteromedial globus pallidus internus (amGPi) has shown sustained significant benefit for Tourette’s syndrome (TS), motor tics and non-motor symptoms such as obsessive-compulsive disorder (OCD), depression, and overall quality of life (Cannon et al., 2012; Sachdev et al., 2012, 2014).

Since 2008 we have performed DBS on 24 patients with severe medically refractive TS often with associated behavioral neuropsychiatric issues. Of these, the first two cases had leads implanted in the posteroventral globus pallidus internus (pvGPi) due to the severity of the motor tics and self-harm. In both of these cases, additional leads were placed in the Nucleus Accumbens (NAcc) to control significant obsessive-compulsive symptoms. The original patient in 2008, prior to DBS, was institutionalized in an adolescent psychiatric facility but required removal of the DBS system in 2016, due to the implanted pulse generator and lead infection. This patient has remained stimulation free now for 5 years. There was significant return of her TS symptoms, but they are living with assistance in the general community.

The third patient had OCD symptoms greater than the motor and phonic tics, and the leads were placed into the NAcc with a good clinical outcome of the OCD, and to a lesser extent motor tics.

The remaining 21 patients had leads placed in the amGPI with significant benefit for TS and OCD symptoms as well as depression and overall quality of life. This benefit has been sustained over the years since these people had their surgery. They have had ongoing follow-up and important to note, minimal to no change in the DBS programming parameters were required beyond a 6–12-month time point post-operatively.

One patient with amGPi leads requested removal of the system as they did not feel any different in themselves, despite the fact they were able to obtain gainful employment and interact face-to-face with the public on a daily basis; they have been lost to follow-up.

Our experience now spanning 13 years, overall has demonstrated significant sustained clinical improvement in the quality of life of TS patients in both the primary TS symptoms as well as in sustained reduction in OCD symptoms, depression and improved quality of life.

We feel it is imperative to help medically refractive TS patients obtain wider access to DBS and we strongly support the aims of The International TS DBS Registry and Database. Large double-blind studies at a Class 1 level would delay this access due to low case rates. This delay would be at the cost of humanitarian benefit for TS patients, careers, and society.

### Updates on Telemedicine for Deep Brain Stimulation Care in China

We recently introduced the advances of remote DBS programming in China. Our team made great efforts to design and to develop this remote programming system, which is now widely used in China. Safe remote communication is our priority. The team employed both software protection and hardware protection into the system. The system consists of a physician terminal, server, and patient terminal and offers personalized management and a user-friendly interface, plus real time video consulting and recording. In addition to DBS, the platform also works for vagus nerve stimulation, sacral neuromodulation, and spinal cord stimulation.

Over the past year, we developed an interactive video acquisition and learning system for telemedicine, which will be critical for remote programming. Specifically, the system can automatically record, provide video instructions, and provide real time interactive guidance, and control quality using advanced artificial intelligence (AI) techniques. It offers a default mode for recording with the help of others and a selfie mode for independent recording. Importantly, the system integrates face changing technology for privacy protection. Currently, we can change face identity while preserving facial movements like blinking. We also showed new advances in Bluetooth real time recording DBS: this technique not only stimulates the brain, but also records local field potential (LFP) activity, electrocardiography data, acceleration, and wirelessly transmits to a smart phone simultaneously.

We believe that the remote programming platform and system is highly innovative and will help to democratize DBS therapy. We expect new challenges in adopting this platform, such as the protection of patient privacy or capturing subtle symptoms from remote sensing.

### Advances in Deep Brain Stimulation in Japan

Automatic DBS optimization may be a future perspective that may potentially improve DBS therapy. It includes two main directions: an automatic optimization of initial DBS settings and an on-demand adjustment of stimulation intensity. We performed a single-center, randomized, double-blind, crossover study to evaluate the performance of DBS programming by a closed-loop algorithm (CLA) using an external sensor-based motor assessment which was compared to a standard of care programming method (SOC) in terms of clinical outcomes and programming burden (Sasaki et al., 2021). Both motor score and sensor-based score were significantly improved by both SOC and CLA settings compared to the baseline. In addition, the programming steps were significantly reduced in the CLA settings compared to those in the SOC. This novel algorithm prospectively estimated the optimal stimulation settings for objective assessments and required minimum clinician involvement. Thus, this could lead to a reduction in the number of steps required to program a device in contrast to previous studies that required the same steps as the conventional monopolar DBS screening. This study was performed in patients with octopolar leads, but this novel DBS programming method may enable automatic programming even in more complex DBS leads, such as directional leads. Indeed, AI may upgrade algorithms that enable automatic programming even in more complex DBS leads, such as in a directional lead (Wenzel et al., 2021).

On-demand adjustments of stimulation amplitude using closed-loop stimulation or adaptive DBS may improve DBS therapy. In Japan, the Percept PC (Medtronic Inc.) was launched in November 2020. It offers adaptive stimulation functionality, which at this time is only approved for use in Japan. We are conducting a data collection study to observe the real-world practicality and performance of an adaptive DBS algorithm in patients with PD to observe the resulting LFP dynamics under adaptive DBS in 10 patients (Oyama et al., 2021). The preliminary data revealed that adaptive DBS using the dual-thresholds mode worked as expected. In addition, we are currently conducting a multi-center, open-label study to compare two different adaptive DBS modes; the single and dual thresholds mode, and a multi-center, randomized study to compare adaptive DBS (jRCT1042200088; jRCT1032210376).

## The Front Line of Cutting-Edge Technologies

### Where Is the Future Going? *It’s About Time*

Recent studies have highlighted that patterned stimulation may engage the nervous system in fundamentally different ways than can be achieved with conventional single-frequency stimulation (Tass and Majtanik, 2006; Mastro et al., 2017; Seier et al., 2018; Lo et al., 2020; Spix et al., 2020; Willsey et al., 2020; Ho et al., 2021; Pfeifer et al., 2021). Coordinated reset stimulation may affect synaptic plasticity and result in long-lasting (after stimulation is turned off) effects (Tass and Majtanik, 2006; Ho et al., 2021; Pfeifer et al., 2021). Spatio-temporal paired pulse stimulation can be used to induce spike timing dependent strengthening or weakening of synaptic connections between brain regions and might be used for therapeutic purposes (Lo et al., 2020). Burst stimulation may enable cell-type specific targeting, as recently shown in rodent models of PD and in thalamic stimulation studies in humans (Mastro et al., 2017; Spix et al., 2020; Willsey et al., 2020). Further, adaptive processes of the nervous system would be expected to respond differently to patterned stimulation than to single-frequency stimulation; for example, potentially avoiding the habituation sometimes seen in DBS for ET (Paschen et al., 2019).

The findings suggest the need for a flexible stimulation system that enables further exploration of current and novel patterns in DBS. Chronos is a new research software from Boston Scientific that utilizes the existing flexibility of the commercially available Vercise Genus™ pulse generators, to satisfy this need. Chronos allows the user to choose on a pulse-by-pulse basis, the polarity, amplitude, pulse-width, inter-pulse interval or rate and the spatial location of stimulation (electrodes) while applying historical stimulation safety limits. Importantly, no new firmware is required. Chronos works with the off-the-shelf rechargeable Vercise Genus pulse generators. The ability of Chronos to shape stimulation in time, complements the already existing capability to sculpt stimulation in space and will enable research on the potential of spatio-temporal patterned DBS.

### Responsive Stimulation for Epilepsy

The Long-Term Treatment of responsive stimulation Trial in epilepsy showed a 75% median reduction in seizure frequency at 9 years (Nair et al., 2020). The RNS System Real World Outcome Study showed accelerated results with patients achieving 67 and 82% seizure frequency reductions at 1 and ≥ 3 years, respectively (Razavi et al., 2020). Interim results from an FDA mandated Post Approval Study reported a median 68% reduction at 1 year (Shin and Morrell, 2020).

The Responsive Stimulation for Adolescents with Epilepsy (RESPONSE) Study will begin enrollment in late 2021. This study will enroll 200 participants aged 12–17. The primary end points are the short-term serious device-related adverse event rate and responder rate at 1 year.

NeuroPace received a 5-year NIH grant for a collaborative effort involving eight U.S. academic centers. Six sites will enroll a total of 20 patients with Lennox-Gastaut Syndrome (LGS) and drug-resistant generalized onset seizures. Two sites will create patient-specific maps of the brain seizure networks, providing insight into how to personalize the treatment for each participant. The IDE based study, once approved by FDA, will evaluate the safety and effectiveness of the RNS System in treating seizures associated with LGS. Experience from the study will inform the design of a future larger clinical study. NeuroPace has received Breakthrough Device Designation status from FDA for the potential use of the RNS^®^ System to treat idiopathic generalized epilepsy (IGE) and plans to pursue a clinical study. NeuroPace introduced several product updates including full body MRI conditional labeling, mobile updating of programming tablets, and the launch of the nSight Platform for streamlined physician review of patient data.

### A Role for Local Field Potentials Signals in Supporting the Objective Guidance of Deep Brain Stimulation Therapy Programming and Titration

Next-generation DBS therapy systems have been developed that deliver both standard electrical stimulation therapy and record chronic LFP data through DBS leads implanted in the brain. Medtronic’s first and second-generation DBS + sensing systems, the Activa™ PC + S and Summit™ RC + S, have been utilized in dozens of investigational studies of neurological disorders characterizing unique biomarkers of brain state changes associated with activities of daily living and disease symptomatic states, and for exploring the application of LFP controls signals in adaptive therapy algorithms (aDBS). More recently the Medtronic Percept™ PC with BrainSense™ technology was approved commercially worldwide, offering the capability to apply LFP sensing for monitoring brain activity under real-world conditions in potentially thousands of new DBS patients each year (Paff et al., 2020; Goyal et al., 2021; Jimenez-Shahed, 2021). Two initial case studies of the Percept™ PC implanted in Parkinson’s disease patients report the key finding that the strength of the LFP signal spectral power in the beta range (e.g., 13–30 Hz) corresponds to akinetic rigidity symptoms and their responses to DBS and medication therapies (Feldmann et al., 2021; Koeglsperger et al., 2021), replicating previous studies using investigational recording configurations (Neumann et al., 2016; Ozturk et al., 2020). Importantly, one multi-center study also demonstrates a high prevalence of detectable LFP beta signals of interest in PD patients undergoing each DBS implant center’s standard of care (Thenaisie et al., 2021), which is consistent with a previous multi-center analysis of data collected using the investigational Activa™ PC + S device. Additional Percept™ PC studies confirm the presence of LFP signals of interest in other approved DBS indications, including generalized dystonia, ET and epilepsy (Fasano et al., 2021; Goyal et al., 2021). Further, the emerging implications from these studies and others in progress strongly suggest a role for LFP signals in supporting the objective guidance of DBS therapy programming and titration (Fasano et al., 2021; Sirica et al., 2021). Moreover, several ongoing industry-sponsored trials are evaluating the safety and effectiveness of LFP-beta controlled aDBS in PD. aDBS is already commercially unlocked in the Medtronic Percept™ PC in Japan, where early published results are promising and continue to build upon the evidence of aDBS patient benefit demonstrated by several previous investigational trials (Little et al., 2013; Arlotti et al., 2018; Velisar et al., 2019; Nakajima et al., 2021). Overall, the recent widespread availability of LFP sensing technology embedded in commercial DBS devices offers unprecedented access to objective real-world data and promises a faster path to personalized care for DBS patients. Nonetheless, this large and growing amount of sensing data now being made available by DBS and potentially other neuromodulation devices highlights a critical need for the development of specialized algorithms, tools and infrastructure in order to provide the most potential benefit for patients (Chen et al., 2021).

## Neuroethics of Neuromodulation “Overseas and Outside the Lines”

DBS and implantable neurotechnologies are transforming into a more globalized phenomenon. While major advancements in the field are occurring, primarily in developed countries, these advancements have spurred bioeconomic dependencies between developed, developing, and non-developed nations. Such multi-national efforts have brought into focus the culturally based distinctions in ethical norms and practices that would govern (and thus either constrain or advance) research and translational enterprises (Shook and Giordano, 2017; Giordano, 2018). This differential permissibility and capability has given rise to growing enterprises—and markets—in research and medical tourism.

Of note is that differing ethico-legal standards, when coupled to incentives for multi-dimensional (e.g., economic, social, political, military) leverage (if not hegemony) may result in incurring concerns about safety and security (DeFranco et al., 2020). Thus, while efforts in DBS are aimed at achieving definable “goods” (e.g., treating disease and injury), it is important to address which “goods” are being posited, and the idiosyncratic as well as systemic benefits, burdens, and risks in and across multinational scales that could most likely be incurred by such use(s) in practice (Giordano, 2015, 2017; DeFranco and Giordano, 2020).

Toward such ends, we propose a paradigm of “biosecurity-by-design,” yoked to a cosmopolitan-communitarian neuroethico-legal approach to accurately assess, depict, and mitigate (if not prevent) probable and possible near- and intermediate term effects of DBS use in various contexts (Lanzilao et al., 2013; Shook and Giordano, 2014; DiEuliis and Giordano, 2021).

### The Risks of Differing Ethics to Public Health and National Security

As innovations in neurotechnology such as DBS continue to advance, the use of DBS in military members should be carefully assessed. While primarily used for restoration of health and function (e.g., PTSD or depression), there is a growing trend toward use of neuromodulation for cognitive or behavioral optimization (Lavano et al., 2018; Cinel et al., 2019; Wu et al., 2021). Studies have shown that the most concerning ethical, legal, and social implications of the use of neurotechnology in healthy individuals involves long term safety, invasiveness, reversibility, data security, device security, and social perceptions (Funk et al., 2016; Emanuel et al., 2021). Global economic and military competition will continue to drive much of the policy conversation, as US competitors such as China pursue military advantage through neurotechnologies in combination with artificial intelligence and machine learning advances (Kania, 2019; Center for Security and Emerging Technology et al., 2020). Thus, there exists a compelling need for early risk assessments that involve subject matter expert input, not only to develop risk mitigation strategies, but to enable the discussion of realistic expectations of what neurotechnology such as DBS can potentially deliver. The Think Tank session provided a robust discussion of these issues and offered some novel considerations of device ownership, regulatory approvals, and the potential inevitability of neurotechnology use outside of the amelioration of disease or medical supervision.

### A Proposed “Internationally Relevant” Neuroethicolegal Framework for Deep Brain Stimulation

DBS has proven to be interactive with, and transformational upon, the self-conceptions and patient self-identity. Medical ethical demands include non-maleficence, beneficence and autonomy. However, these retrospective criteria, while necessary, are not sufficient for brain interventions such as DBS to be able to affect prospective agency: one’s ongoing self-conception and self-determination. DBS can be implicated with transforming identity. Narrative ID is about “Who am I becoming?” and possible self-estrangement whereas relational ID is about “How will I be conducting myself?” and the future potential for social estrangement. To address implications for patient identity and autonomy, we must keep in mind how agency and autonomy are generally not considered neurological or physiological matters. DBS cannot be applied in a “medically neutral” environment. The social surroundings and cultural traditions will impact DBS. Questions must be asked to challenge culture-bound presumptions. How do people experience the application of DBS in the ongoing course of their lives within their own social groups? How do people assess DBS’s value for themselves, in terms of their healthcare needs and their mutable self-conceptions and values? How has the application of DBS for members of society been evaluated for responsible innovation, genuine social need and justice? How does a culture generally assess DBS’s impacts on people’s lives, according to customary values, cherished ideals, and established laws? In general, a brain cannot determine the self, since self-conceptions are tied to social capabilities. Neuroethics should not presume that one nation’s culture holds unique standards for mental health, responsible agency and good character.

## Interventional Psychiatry: Updates From the NIH Brain Initiative

### Non-linear Recovery in Electroencephalography and Fine-Grained Behavior During Subcallosal Cingulate Deep Brain Stimulation for Depression

Evidence from studies of subcallosal cingulate (SCC) DBS for treatment resistant depression demonstrate that antidepressant effects occur in two stages: a rapid change in negative mood and psychomotor slowness with initial bilateral stimulation at the optimized target within the SCC white matter, and a slower progressive improvement in symptom ratings over weeks to months that if achieved, is generally maintained long-term. Combined behavioral, imaging, and electrophysiological strategies may facilitate a more fine-grained characterization of this chronology. We describe our strategic acquisition of qualitative and quantitative behavioral measures with LFP recordings suitable for both direct hypothesis testing and for unsupervised machine learning approaches. Building on previous experiences using weekly standardized ratings and video analyses of weekly clinical interviews, our studies capitalize on twice daily sampling of SCC LFPs, self-report behavioral ratings, online depression severity scales and video diaries with concurrent SCC LFPs. These results demonstrate that there are meaningful behavioral features that track with acute and chronic brain changes, potentially enabling the future development of clinically tractable biomarkers that can be used to guide therapy.

### New Data-Driven Electrophysiology Outcome Measures and Insights Into Subcallosal Cingulate Cortex Deep Brain Stimulation for Depression

The SCC has been an effective target for DBS in treating patients with treatment-resistant depression, but individual patients exhibit high variability in recovery trajectories. Understanding the changes in neural activity underlying sustained recovery will help us to identify a physiological marker to track this variability in recovery trajectories. The marker is also particularly relevant in the context of adaptive neurotechnologies for CL stimulation. The increasing interest in adaptive neuromodulation has led to the collection of large amounts of multi-modal data, as well as to the application of machine learning (ML) techniques to provide insight. In conventional ML approaches, there is typically a tradeoff between complexity and interpretability: simple models can be interpretable but capture only rudimentary structure in the data, while complex “black-box” models can capture more intricate relationships at the expense of interpretability. Recently, the framework of “explainable artificial intelligence (xAI)” has introduced approaches that aim to explain these powerful “black-box” models, making them especially suited for identifying biomarkers. During this Think Tank, we discussed an example of using generative causal explainers (GCE) to analyze an existing machine learning network and its associated output data (O’Shaughnessy et al., 2020). The GCE is a type of neural network that will capture features from an existing ML network and identify a spectral discriminative component (SDC). This SDC represents the relationships between the original training data and output that were not provided by the original machine learning model and can then be used to identify potential biomarkers. We have collected LFPs from six participants undergoing SCC DBS who showed variable recovery trajectories preceding robust therapeutic response at the 24-week endpoint. We used recently developed techniques from xAI to show that meaningful objective markers of disease state can be extracted from LFP data that correspond to independent behavioral and anatomical measures. These results demonstrate the potential for xAI techniques to be used to develop biomarkers in complex neuromodulation therapies.

### Combined Cortical and Subcortical Recording and Stimulation as a Circuit-Oriented Treatment for Obsessive-Compulsive Disorder

OCD is associated with hyperconnectivity in a specific cortico-striato-thalamo-cortical (CSTC) circuit including the orbitofrontal cortex (OFC), the head of the caudate and the dorsomedial nucleus of the thalamus. Traditional DBS at the FDA-approved ventral capsule/ventral striatum (VC/VS) target for intractable OCD is believed to exert its beneficial effects by disrupting this hyperconnectivity. Here we present a case report of an attempt to desynchronize this CSTC circuit using multi-site stimulation: the standard VC/VS target plus bilateral cortical leads at the supplementary motor area (SMA). Clinically, the patient’s Yale–Brown Obsessive Compulsive Scale (YBOCS) score decreased from the low 30’s to 16. There was immediate subjective improvement, with a sense of ability to focus away from obsessions, without the mirthful or anxiolytic qualities of VC/VS stimulation. This took years to be reflected in the YBOCS. Physiologically, there was a hyper-synchronized peak in the high alpha band in both acute intraoperative and long-term Medtronic PC + S recordings. It was stimulation sensitive, but contrary to the initial model, synchrony increased over time, and higher synchrony reflected clinical improvement. These preliminary findings suggest that multi-site stimulation may be effective for treating intractable OCD. Electrophysiological biomarker changes may be associated with this improvement. These changes may reflect a DBS mechanism where hypo-functioning CSTC loops are augmented, rather than a disruption of a hyper-connected loop.

### Deep Brain Stimulation for Depression Using “Inverse Solutions” Enabled by Intracranial Recordings

Current biological views suggest that (1) disorders of mental health are network disorders, and (2) that they therefore demand network-minded solutions. With this idea in mind, we embarked on our NIH-funded (UH3 NS103549) trial (NCT03437928) of DBS for treatment-resistant depression (TRD). Our approach borrows the platform of intracranial recording and stimulation which is common to the field of epilepsy surgery (Allawala et al., 2021). We recruited patients with severe TRD implanted with bilateral DBS leads in both sub-callosal cingulate (SCC) and ventral capsule/ventral striatum (VC/VS) targets. In addition, we implanted stereo-EEG (sEEG) electrodes in regions across the putative frontotemporal network which were relevant to TRD (Vedam-Mai et al., 2021). This strategy facilitated recording from mood-relevant areas during rest, during specific activities and behavioral tasks, and while delivering stimulation across a wide range of parameter space. Following this inpatient phase, the sEEG electrodes were removed, and the patient continued in an outpatient trial consisting of an 8-month open label optimization phase followed by a randomized, double-blinded discontinuation phase.

**FIGURE 4:**
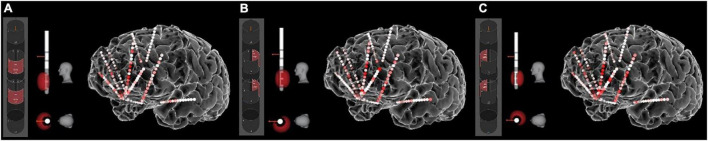
Stimulation-induced network states. The sEEG recordings allow us to measure the “neural state” produced by any particular set of stimulation parameters. Here the heat map shows gamma (40–70 Hz) power in response to stimulation of various contact combinations on the left SCC DBS lead. **(A)** Contact configuration 2–5 (anterior stack of segmented contacts). **(B)** Contact configuration 3–6 (posterior-left stack). **(C)** Contact configuration 4–7 (posterior-right stack). Other parameters were frequency of 130 Hz, pulse width of 180 ms, and amplitude of 5 mA. Other combinations of these parameters produce different neural states, all of which can be quantified with the sEEG recordings.

An important innovation in this trial was the use of intracranial electrophysiology data to calculate “inverse solutions”: DBS parameters that are calculated to produce a healthier brain network state (as measured by neural activity on the sEEG electrodes) and therefore to reduce symptoms. Our first attempt at this calculation involved a template-matching process. We determined a desirable network state based on both spontaneous mood changes and mood changes induced by a behavioral task. We also measured network states produced by stimulation across many parameter combinations (Figure 4). We then used an iterative general linear model to identify parameter combinations that best matched the desired state. The first subject in this trial achieved remission that was robust to the double-blinded discontinuation, suggesting true rather than sham response to DBS.

## Advances in Adaptive Deep Brain Stimulation

### Optimizing Subthalamic Adaptive Deep Brain Stimulation for Parkinson’s Disease

**FIGURE 5:**
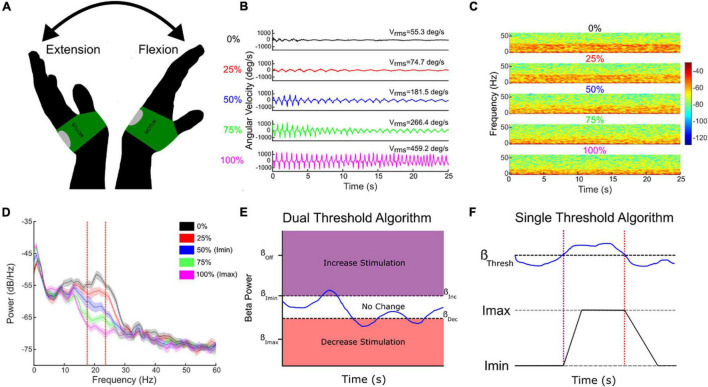
Individual randomized presentations of STN DBS intensity, normalized to the maximum tolerated without side effects (Imax, 100%, pink traces), during a repetitive wrist flexion extension task **(A,B)**, determine the safe and acceptable range (Imin (here 50% Imax) to Imax), through which DBS intensity will fluctuate **(B–E)**. Corresponding beta power measured at Imin and Imax determine the upper and lower beta thresholds for the dual threshold algorithm **(D,E)**, and the beta power for the single threshold algorithm **(F)**. Blue line **(E,F)**—fluctuating beta power.

Successful aDBS in neurological diseases requires inputs that are relevant to the behavior targeted for therapy. Although Subthalamic Adaptive Deep Brain Stimulation (STN aDBS) has been shown to be safe, feasible, efficacious and more efficient than open loop or continuous (c)DBS, several variables remain to be optimized (Little et al., 2013, 2016a,b; Rosa et al., 2015; Malekmohammadi et al., 2016; Cagnan et al., 2017; O’Day et al., 2020). The STN alpha and beta LFP power spectrum usually comprises more than one band and one of these may demonstrate more attenuation from STN DBS than the other (Figure 5D; Shreve et al., 2017; Afzal et al., 2019). The beta band with the greatest power has been the usual neural input and we have shown that aDBS driven by either the modulated or unmodulated band was efficacious (Afzal et al., 2019). Both dual and single threshold control policy algorithms are feasible (Figures 5E,F), and depend on choosing beta thresholds that correspond to the minimum and maximum DBS intensities (Imin, Imax) that provide acceptable therapy, which we determine using individual titrations of DBS intensity during movement (Figure 5; Little et al., 2013; Velisar et al., 2019; Kehnemouyi et al., 2021). Other relevant neural inputs include prolonged beta burst durations, which are related to disease severity, bradykinesia, and freezing of gait (FOG) (Tinkhauser et al., 2017; Anidi et al., 2018; Kehnemouyi et al., 2021). aDBS can also be driven by relevant behavioral inputs, such as tremor intensity or gait kinematics (Malekmohammadi et al., 2016; Cagnan et al., 2017; Herron et al., 2017; O’Day et al., 2020; Diep et al., 2021). The goal of therapy will determine the rates, at which DBS intensity is adjusted: slow ramps will adjust DBS intensity based on the time course of the onset and offset of medication doses, whereas faster ramps may respond to beta burst durations, and stochastic events such as tremor or FOG (Arlotti et al., 2018; Petrucci et al., 2020a,b, 2021). These therapy values can be determined on an individual basis.

### Adaptive Deep Brain Stimulation in Parkinson’s Disease: Technical Considerations and Lessons Learned

**FIGURE 6:**
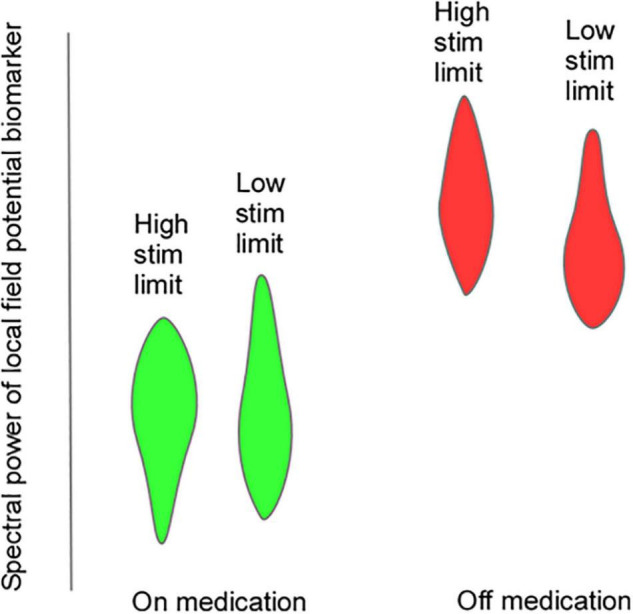
Hypothetical violin plots showing distribution of a putative LFP biomarker that would be useful for “slow” aDBS. The biomarker distributions distinguish on-medication (green) and off-medication (red) states at both upper and lower stimulation amplitude limits.

Efforts to develop aDBS in PD have focused on two different strategies: (1) Detection and truncation of pathologically prolonged bursts of beta oscillatory activity, on a time scale of seconds (“fast” aDBS). (2) Detection of neurophysiologic signatures of medication “on” and medication “off states, with concomitant decreases and increases in stimulation amplitude on a time scale of minutes to hours (“slow” aDBS). A challenge with fast aDBS is that rapid changes in stimulation have been associated with brief electrical artifacts whose spectral signature is broadband. Thus, rapid down-ramping at the end of a beta burst may produce an artifactual detection of elevated beta activity, triggering an inappropriate increase in stimulation even in the absence of underlying beta bursting. Mitigation strategies include blanking of sensing during an interval after stimulation ramp-down and reducing the difference between stimulation amplitude limits. A challenge of slow aDBS is that many empirical iterations of aDBS parameters may be required for personalized optimization. We have developed a “principled” approach for rapid prototyping of adaptive control policies for slow aDBS. First, we identify upper and lower stimulation limits corresponding to a patient’s needs in different medication states, in the clinic setting or at home. We stream time series neural activity at those two stimulation amplitudes for several medication cycles. We then plot the statistical distribution of spectral power at each frequency band up to 90 Hz, and identify bands that best distinguish medication states, and can do so regardless of stimulation amplitude (Figure 6). These frequency bands or band combinations are then used in a dual threshold control policy with lower and upper thresholds determined by percentiles of the biomarker distributions.

### Using Physiology to Drive Tremor Suppression

ET is defined as a rhythmical, involuntary, oscillatory movement of the limbs and is one of the most common movement disorders DBS has been an effective therapy for the suppression of medically refractory tremor. However, as intention tremor occurs mostly in the upper limbs during the initiation and execution of goal-directed reaching motions, while it is absent at rest, continuous stimulation (cDBS) is in large part unnecessarily delivered, consequently leading to inefficient therapy and unneeded potential DBS-induced side-effects. An aDBS approach facilitates targeting a direct or indirect neuromarker(s) of reference, to deliver stimulation only when the patient truly needs it (e.g., during movement).

We established the feasibility of behavior-based aDBS for ET, fully embedded in a chronic investigational neurostimulator (Activa PC + S), for three patients implanted with a VIM-DBS, enrolled in a longitudinal (6 months) within-subject crossover protocol (DBS OFF, cDBS, and aDBS). As tremor manifests once movement is initiated, we explored the efficacy in modulating the stimulation amplitude based on the cortical motor activity of the patient’s upper limbs. The proposed aDBS paradigm resulted in clinical efficacy and tremor suppression comparable with cDBS within a range of common actions (cup reaching, proximal and distal posture, water pouring, and writing), with a considerable reduction of stimulation delivered, showing the potential for integrating DBS therapy with the patient behavior and for potentially addressing pitfalls of cDBS for ET, such as DBS-induced side effects and premature device replacements.

## Neuromodulation for Pain

### Real-Time Evoked Compound Action Potential Controlled Closed-Loop Spinal Cord Stimulation

Evoked compound action potential (ECAP) recording provides an objective measure of spinal cord (SC) activation during spinal cord stimulation (SCS) and can assist in programing of the SCS system. The Evoke Study Group conducted a double-blind randomized controlled trial (RCT) to compare the safety and efficacy of real-time ECAP-controlled closed-loop stimulation (investigational group) with open-loop (fixed output) stimulation (OL, control) to treat chronic back and leg pain.

**FIGURE 7:**
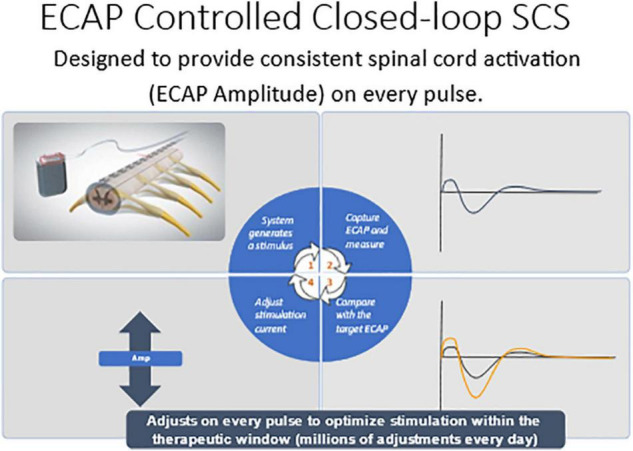
Computer controlled ECAP controlled closed-loop SCS automatically adjusts current output to maintain a constant ECAP amplitude.

There were 134 subjects enrolled and randomized after test trial leads were implanted. The target ECAP amplitude was recorded on the same lead as the stimulating electrode was set in the clinic and it was maintained either manually by the patient (OL) or by a computer-controlled feedback closed-loop control (CL) mechanism (Figure 7). The primary endpoint evaluated as ≥ 50% reduction in overall back and leg pain as measured by the Visual Analog Scale (VAS). Opioid usage and other patient-reported outcomes (PROs) including emotional/physical functioning, sleep quality, and quality of life were also collected. Additionally, objective neurophysiological data, including SC activation and time spent in the therapeutic range, were collected.

**FIGURE 8:**
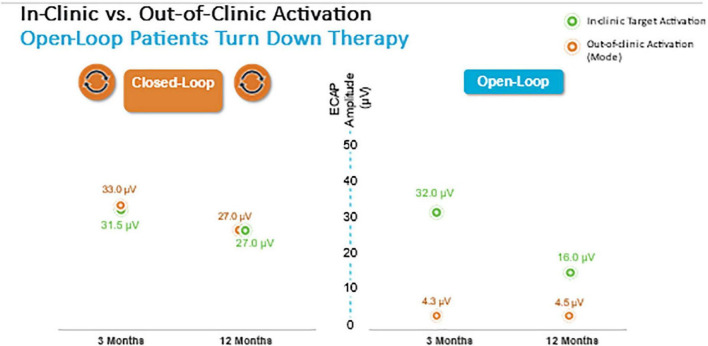
Closed-loop but not Open-loop group was able to maintain the target ECAP level in the clinic and well as in the out of clinic environment.

Herein the Evoke Study Group reports the 24-month outcomes from this ongoing RCT. The proportion of implanted subjects with ≥ 50% overall back and leg pain reduction at 24 months was statistically superior in the EVOKE CL vs. OL group (84.0 vs. 65.9% subjects, respectively; *p* = 0.040) (Figure 3). Long-term improvements in all other PROs, including Profile of Mood States, Oswestry Disability Index, Pittsburgh Sleep Quality Index, EuroQol quality of life (EQ-5D-5L), and the Short-Form Health Survey (SF-12), were also demonstrated. In addition, the patient/physician identified the target ECAP amplitude during programming that was the same for both groups, however, the OL group was unable to maintain this ECAP target in the outpatient setting (Figure 8). The most frequent level of SC activation was three times greater for CL (median ECAP Amplitude: 22.5 μV CL vs. 7.5 μV OL).

SC activation was better maintained within the therapeutic range with EVOKE CL (median: 93.9% CL vs. 46.1% OL). There were no differences in the safety profiles between treatment groups, and the type, nature, and severity of adverse events were similar to other SCS studies.

In this ongoing study, ECAP-controlled closed-loop spinal cord stimulation provided statistically superior pain relief and greater improvement in all other measures compared with the OL group at 3, 6, 12, and 24 months. This significant improvement in clinical outcomes was consistent with the CL group being able to better maintain the targeted spinal cord activation level as measured by the stability in the ECAP amplitudes.

### Personalized Circuit Mapping and Deep Brain Stimulation for Pain

Pain is the most fundamental human experience yet understanding of basic brain mechanisms relevant to human disease has been elusive. Following the workflow for refractory epilepsy, we proposed trialing brain stimulation and recording through temporary placement of invasive electrodes to identify therapeutic neural targets for each patient. After such a trial, we have been able to achieve enduring pain relief for research subjects by targeting these brain regions with a permanent DBS system. Although this process was labor intensive and economically more challenging, this approach could provide flexibility needed to address the wide heterogeneity observed in individual brain function during pain. Fundamental questions regarding optimizing brain stimulation for chronic pain remain. Is experimentally induced pain supported by similar brain circuits as a legitimate chronic pain syndrome(s)? If a brain region harbors signals important for decoding an individual’s pain state, can stimulating this same region modify pain perception? While working toward answers to such questions, some early clues have emerged. The research community should be more sensitive to quantitative measures of pain that have a wide dynamic range within subjects. Brain-based neurophysiology methods such as intracranial recording and electroencephalography (EEG) may help to better characterize clinical pain phenotypes. Finally, to avoid the long-term loss of therapy that has plagued many prior DBS efforts for pain, it may help to limit the cumulative electrical dosage by using adaptive stimulation paradigms. Solving these critical issues will aid in the development of new options to treat refractory chronic pain.

## Network Modulation for Drug Resistant Epilepsy

### Drug Resistant Epilepsy: Generalized and Focal Epilepsy Networks

**FIGURE 9:**
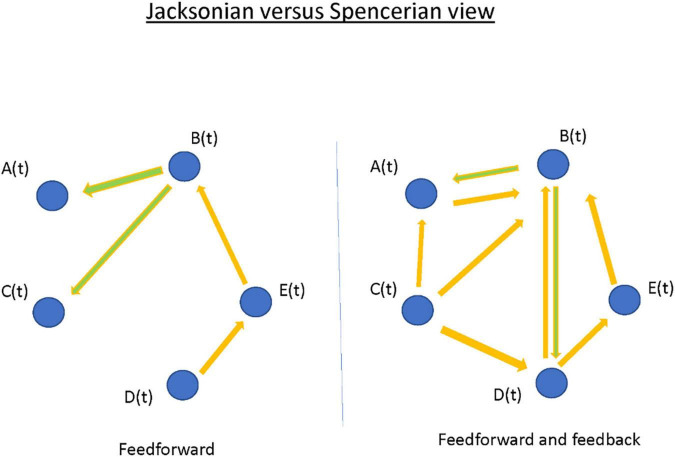
Network propagation models of partial epilepsy syndromes.

Positing focal epilepsy as a “disorder of brain networks” has several interpretations. In one sense, the comorbidities associated with chronic partial epilepsy—memory dysfunction, or anxiety and depression—imply compromise of functional networks involved in integrated cortical action. In another and more immediate sense, the phenomenon of the seizure itself is proposed as a collective property of multiple, non-contiguous, brain areas. The latter view, often traced back to Spencer, seemingly countermands the traditional (“Jacksonian”) view of a partial seizure having a delimited “focus” that recruits other brain areas by propagation (Figure 9; Spencer, 2002). I argue that the Jacksonian and Spencerian views are not mutually exclusive. I describe three cases of focal epilepsy, all investigated by invasive EEG (stereo-electroencephalography; SEEG), where seizures were observed to behave at either extreme and/or at an intermediate level. More abstractly, I propose the Jacksonian view as modeled by a network with feed-forward connections only, such that information (i.e., seizure) flows along a definite causal path. In contrast, the Spencerian view is modeled by both feedforward and feedback connections, where coupling between nodal network elements makes the question of an “initiating” focus and the direction of its propagation irrelevant. I speculate all focal epilepsies fall within the spectrum defined by these extremes. Finally, I outline the analytical challenges in understanding multivariable interacting dynamical elements such as in the proposed conceptualization. However, rational choices for surgical therapies and neuromodulatory targets for the most difficult patients may depend on scientific progress.

### Reassessing the Purpose of Stereoelectroencephalography

During surgical treatment and assessment for epilepsy, patients are considered in multidisciplinary epilepsy conference (MDEC) by a team of neurologists, neuroradiologists, psychiatrists, neuropsychologists, and neurosurgeons. The patients’ symptoms surrounding seizure (semiology), brain imaging (abnormality = “lesional”), and scalp electroencephalography are compared for agreement (concordance) by the team. In the case of concordance indicating seizure initiation from a non-eloquent brain site, we proceed to resection to cure the epilepsy. In cases of indeterminate concordance surgical implantation of monitoring intracranial electrodes, including brain-penetrating, Stereoelectroencephalography (sEEG) electrodes may further localize the seizure onset. The current paradigm is to place each sEEG lead in a candidate brain region to identify (or to rule out) a resectable seizure onset zone. However, the likelihood of ultimate resection with excellent outcome (Engel 1) for a patient considered in MDEC can be as low as 10–20%, with the likelihood increasing to ∼30–40% for those who undergo intracranial monitoring (about ∼50% of the ∼60% who proceed from intracranial monitoring to resection) (Téllez-Zenteno et al., 2005; Noe, 2013; Jehi, 2015). For patients who do not undergo resection, or those with persistent seizures refractory to resection, implanted brain stimulators are an important palliative option for potential reduction of seizure frequency and intensity.

**FIGURE 10:**
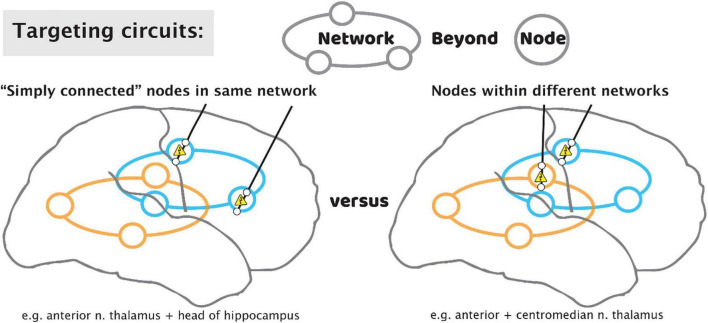
Targeting circuits with stimulating leads for epilepsy. As we move beyond a paradigm that focuses solely on the seizure focus, approaches may include targeting of multiple “simply connected” nodes in the same network **(left)**, or tandem nodes within different networks **(right)**.

Brain stimulation for epilepsy currently falls into two general paradigms: The first of these is stimulation of the electrophysiologically-identified seizure onset zone(s) (SOZ)—the node(s) idiosyncratic to each patient’s epilepsy. This stimulation with a “customized construct” may be responsive to detected electro-graphic activity, or it may be OL with pre-set stimulation parameters (Morrell, 2006). A second paradigm is to stimulate additional network nodes in the seizure circuit that are not at the SOZ but is a common site of confluence. These sites will be the same across different patients, with a “general construct,” and typically target a thalamic nucleus. To date, the anterior nucleus of the thalamus (ANT) is the only location to be assessed through an FDA premarket approval clinical trial (Fisher, 2010; Salanova, 2021). However, one size does not fit all when it comes to thalamic nuclei, and the ANT, a component of limbic circuitry, is not a universal node in all seizure networks. Epilepsies come from discernible networks, so the nucleus of thalamic stimulation should be determined by the putative network involved. The centromedian nucleus is suggested for basal-ganglial, motoric, and generalized epilepsies; the pulvinar has been suggested as a common target for occipital-onset seizures, and those with eye movement semiologies; the central lateral (intralaminar) nucleus is being trialed for non-lesional, extratemporal epilepsies of impaired awareness; further such targets will be identified based upon the evolving neuroscientific understanding of how the hemispheres interact with the thalamus (Gummadavelli, 2015; Warren, 2020; Burdette, 2021). We propose that each patient’s epilepsy should be characterized for distinct thalamic and hemispheric nodes for potential stimulation, depending on semiology, electrophysiology (ictal and inter-ictal), and stimulation during sEEG monitoring (test stimulation and stimulation evoked potentials). As we move to more optimized stimulation constructs, we will move beyond a “node-based” philosophy, toward a “network-based” philosophy, using patient-specific diagnostics to stimulate multiple nodes within and across networks (Figure 10).

Because most patients will not progress to an excellent outcome by way of resection, we propose that the role of sEEG should be elevated to emphasize optimization of subsequent stimulator implant in addition to the traditional role of identifying resection regions. After the period of diagnostic passive monitoring for seizure localization, test stimulation through the sEEG leads may be performed, monitoring the patient for reduction in seizures or a reduction in interictal epileptic spiking rate (Lundstrom, 2019). A trial of CL sense-and-stim during this period is also possible (Kossoff, 2004). Single-pulse electrical stimulation is an emerging tool to study interactions between network nodes, with new insight(s) enabling simplifying interpretation circuit electrophysiology (Miller et al., 2021). Informed placement of thalamic electrodes may be performed to understand stimulation targets and seizure propagation, though these sites are not a potential resection target. Informed by these thalamic recordings, we may leverage sEEG studies to place tandem leads aiming for nodes that are “simply connected” within the same circuit (Gregg, 2021), or nodes in distinct circuits for a broader effect in seizure suppression (Figure 10).

## Neuromodulation for Traumatic Brain Injury

Traumatic Brain Injury (TBI) is a leading cause of long-term disability, due in large part to a lack of effective treatment options. Successful treatment of impaired mental processing speed and executive function could improve patient quality of life. Converging evidence from prior work in rodents, non-human primates and humans has provided evidence to support improving arousal and cognition through stimulation of central thalamus. We initiated a 6-participant feasibility study (CENTURY-S, NCT02881151, funded by NIH BRAIN Initiative grant UH3 NS095554) of central thalamic DBS (CT-DBS) in patients with moderate to severe TBI (msTBI). The trial was based on the hypothesis that activation of down-regulated frontostriatal systems would improve cognitive dysfunction, increase information processing speed and decrease fatiguability. We have reported our preliminary findings on CT-DBS in five participants to date with longstanding functional disability related to persistent cognitive dysfunction after severe TBI (age 23–60, 3–18 years after injury).

**FIGURE 11:**
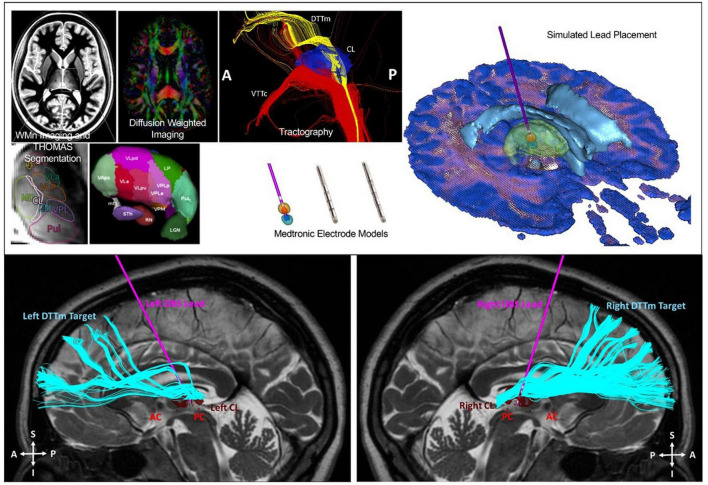
Top image: Identification of the CL and DTTm. Automated patient-specific thalamic segmentation was performed to obtain detailed representations of the central lateral (CL) nucleus for use as a spatial filter to identify the DTTm projecting from the brainstem to the frontal cortex. Bottom image: Two Medtronic 3389 4 contact DBS leads were implanted into the CL nucleus under monitored anesthesia care with microelectrode mapping. The leads were attached to a right infraclavicular Activa PC + S pulse generator under general anesthesia in the same surgical session.

Six patients underwent implantation of bilateral electrodes into the central lateral (CL) nuclei, specifically targeting the medial dorsal tegmental tract (DTTm) guided by diffusion tensor imaging tractography and a participant-specific map of the thalamus generated by the THOMAS thalamic segmentation pipeline (Figure 11). CL and other thalamic nuclei were identified using the THOMAS atlas template in combination with a white matter nulled image sequence (Tourdias et al., 2014; Su et al., 2019). The DTTm was identified using tractography seeded from CL and the pedunculopontine nucleus. Avoidance fiber tracts were identified by seeding the centromedian (CM) and mediodorsal (MD) thalamic nuclei. The virtual DBS platform was used pre-operatively to explore and to select the DBS lead trajectory in each hemisphere (Janson and Butson, 2018). This plan was imported into the surgical planning system. The 30-day post-operative computed tomography imaging was used to determine actual lead location after being registered to the pre-operative T2 MRI, which was used as the base image for surgical planning and patient-specific modeling. This updated model was used to guide post-operative selection of DBS parameters. We have successfully used this approach to implant DBS leads bilaterally in six study participants to date. One participant was explanted due to infection and was not reimplanted; five subjected therefore received stimulation during the open-label period with four having completed the full treatment phase. Two subjects were randomized to a blinded withdrawal at treatment end and two refused randomization due to a perceived therapeutic effect that was lost with brief stimulator deactivation (one intentional, one accidental) during the open-label period.

The study design included a 2-week stimulation titration phase (TP) and a 3-month open label treatment phase. All participants completing the treatment phase to date met the pre-selected primary outcome benchmark of a greater than 10% improvement in completion time on the Trail-Making Test part B (TMT-B) from pre-surgical baseline to the end of the TP (median = 24.84%; IQR: 21.8–32.2). On the TBIQoL-Fatigue measure, one participant of the four met the improvement benchmark, two remained stable and one met the benchmark for decline (although this single measurement was obtained during an intercurrent viral illness). The improvement in processing speed observed on the TMT (A and B) was concordant with the self-reported improvement noted on the TBIQoL-Attention measure. Despite the short 3-month open label phase, two of the four subjects who completed the trial showed a 1-point increase in the GOS-E rating from the presurgical baseline to the end of the TP. These findings preliminarily demonstrated the safety of implantation, evidence for improved mental control under speeded conditions and resistance to fatigue.

Our primary rationale was to match the underlying pathophysiological substrate of chronic cognitive impairment in patients with severe to moderate TBI to the use of CT-DBS as an intervention. The severity of initial overall cerebral deafferentation as indexed by clinical variables was linked to neuropsychological measures of working memory, learning, attention, and information processing speed deficits after msTBI (Dikmen et al., 2003). The central thalamus is anatomically specialized to provide strong synaptic drive across the frontal (particularly medial frontal) and prefrontal cortices and rostral striatum in response to cognitive demands that support these “executive functions” (Liu et al., 2015; Baker et al., 2016). CT-DBS was proposed to activate these systems sufficiently to provide effective functional improvements. Our group previously carried out a first-in-human study of CT-DBS in subjects with very severe traumatic brain injuries. In one subject studied, we established that the recovery of spoken language, deglutition, and executive functions (including but not limited to attentive behavioral responsiveness, motor executive control and access of episodic memory) 6 years following injury was causally linked to CT-DBS (Schiff et al., 2007).

Electrical stimulation of the primary CL lateral wing cell bodies/DTTm fibers was not associated with abnormal sensations or movements. Regions more ventral to CL/DTTm elicited transient side effects in patients including speech slurring, jaw sensations and perseveration; these effects may relate to activation of the centromedian-parafasicularis (Cm-Pf) and more medial components of the median dorsalis (MD) nucleus, respectively. There was marked improvement in performance on the primary outcome measure from the pre-surgical baseline to the end of the 90-day treatment phase for all four subjects completing the study (to date).

This is the first study of DBS electrode implantation in moderate to severe traumatic brain injury with subsequent recovery (outcome range of GOSE 5–7) to remediate impaired cognitive function. The generalizability of these findings will require testing in a larger sample.

## Summary and Conclusion

**FIGURE 12:**
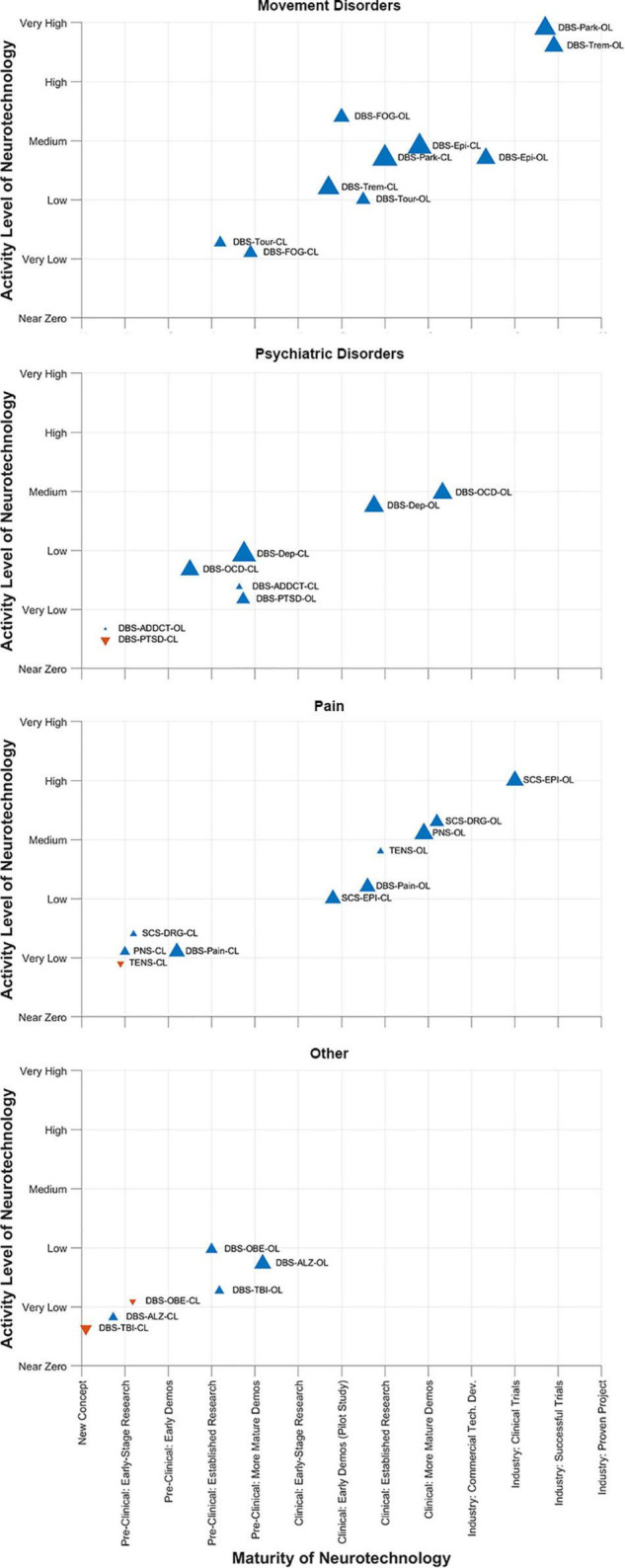
DBS-Think-Tank Neurotechnology Activity-Maturity Graphs. This figure presents four graphs that illustrate the perceptions of DBS-Think-Tank attendees about the maturity, activity, and change in activity of a variety of neurotechnologies. The neurotechnologies are organized into the following four groups and graphed separately: movement disorders, psychiatric disorders, pain disorders, and other syndromes. The upward pointing blue triangles represent increasing activity and downwards pointing orange triangles represent decreasing activity. The magnitude of the change is proportional to the size of the triangles. The definitions of the abbreviations used to identify each triangle are as follows: DBS, deep brain stimulation; OL, open loo; CL, closed loop; Park, Parkinson’s disease; FOG, freezing of gait; Epi, epilepsy; Trem, tremor; Tour, Tourette’s syndrome; OCD, obsessive-compulsive disorde; Dep, depression; PTSD, posttraumatic stress disorder; ADDCT, addiction; PNS, peripheral nerve stimulation; TENS, transcutaneous electrical nerve stimulation; Pain, chronic pain; DRG, dorsal root ganglia stimulation; SCS, spinal cord stimulation; ALZ, Alzheimer’s disease; OBE, obesity; TBI, traumatic brain injury. The data presented were derived from survey respondents with clinical, scientific, engineering, and commercial expertise and had academic, industrial, government, and non-profit professional backgrounds.

This year, the DBS Think Tank IX advances focused on cutting-edge technologies and the use of novel methodologies for tracking and suppression of symptoms. The neuroethics of neuromodulation session focused on international issues, device security and a path forward. The DBS Think Tank group agreed that a brain cannot determine the self, since self-conceptions are tied to social capabilities. Neuroethics should not presume that one nation’s culture holds unique standards for mental health, responsible agency and good character. This year we saw exciting growth in DBS for depression, pain, epilepsy and TBI. Advances in the application of physiology and imaging have driven novel indications and the hope is that these technologies will also drive improved outcomes. Investigation of neurophysiological signals in neurological disorders continue to explore new biomarkers and involved networks potentially amenable to neuromodulation. The CL physiology approaches to DBS will likely present important barriers to implementation and the programming strategies will likely be highly individual. It was not clear to the DBS Think Tank group that CL DBS would be effective for all disease indications. Interactive video acquisition and facial recognition was considered as possible biomarkers for depression and other diseases and also was considered for use in telemedicine adjustments for DBS. The neurotechnology “hype curve” for these topics are shown in Figure 12.

This year the DBS Think Tank IX also discussed self-actualization. Many people have heard of Maslow’s hierarchy of needs, but most of what people know about his famous “pyramid” is wrong (Maslow, 1943). Briefly, the hierarchy of needs describes five levels of human needs. From bottom to top, the levels are physiological needs, safety needs, love and belonging needs, esteem needs, and the topmost layer of self-actualization. The underlying model posits that one cannot attend to needs of a higher level until the needs of a lower level have been satisfied. This concept is often depicted as a pyramid with self-actualization positioned at the top. However, Maslow did not draw a pyramid. He was suggesting a journey toward self-actualization, but did not limit his concept to a simple pyramidal structure. We discussed both the person with an implanted device and the clinician-scientist-engineer-researcher’s journey toward self-actualization and eventually transcendence. We discussed Dr. Kaufman’s research placing the hierarchy of needs on a more stable scientific foundation and we examined the list of the characteristics of self-actualizing people. This work has provided a revision of the hierarchy of needs. This revision draws on the science of creativity, on love and on transcendence. Using this revision, we can apply self-actualization and transcendence to the community of people involved in brain implant technology development and deployment, as well as to the journey of the patient and caregiver. The integration of these concepts has the potential to be important for people in the implantable device arena as well as other areas of medicine. It can help others reach higher states of consciousness while maintaining agency despite an implantable device being present in the body.

## Data Availability Statement

The original contributions presented in the study are included in the article/supplementary material, further inquiries can be directed to the corresponding author/s.

## Ethics Statement

The studies involving human participants were reviewed and approved by the individual academic institutions. The patients/participants provided their written informed consent to participate in this study.

## Author Contributions

All authors listed have made a substantial, direct, and intellectual contribution to the work, and approved it for publication.

## Conflict of Interest

RR was employed by Medtronic, Inc. HBo was employed by Boston Scientific Neuromodulation Corporation. DG was employed by the NeuroPace, Inc. The remaining authors declare that the research was conducted in the absence of any commercial or financial relationships that could be construed as a potential conflict of interest.

## Publisher’s Note

All claims expressed in this article are solely those of the authors and do not necessarily represent those of their affiliated organizations, or those of the publisher, the editors and the reviewers. Any product that may be evaluated in this article, or claim that may be made by its manufacturer, is not guaranteed or endorsed by the publisher.
